# Prognostic evaluation of febrile neutropenia in apparently stable adult cancer patients

**DOI:** 10.1038/bjc.2011.284

**Published:** 2011-08-02

**Authors:** A Carmona-Bayonas, J Gómez, E González-Billalabeitia, M Canteras, A Navarrete, M L Gonzálvez, V Vicente, F Ayala de la Peña

**Affiliations:** 1 Department of Haematology and Medical Oncology, University Hospital Morales Meseguer, Avda Marqués de los Vélez, S/n 30001, Murcia, Spain; 2 Department of Infectious Diseases, Hospital Virgen de la Arrixaca, Carretera de Madrid-Cartagena, S/n 30120, Murcia, Spain; 3 Department of Biostatistics, Faculty of Medicine, University of Murcia, Campus de Espinardo, 30100, Murcia, Spain; 4 Department of Medical Oncology, Hospital Virgen de la Arrixaca, Carretera de Madrid-Cartagena, S/n 30120, Murcia, Spain

**Keywords:** febrile neutropenia, apparently stable patients, prognostic model

## Abstract

**Background:**

Predictive models to identify low-risk febrile neutropenia (FN) have been developed with heterogeneous samples, which included stable and unstable patients, solid tumours, acute leukaemia and bone marrow transplantation. These models fail to recognise 5–15% of cases with unexpected complications, and literature specifically addressing apparently stable patients (ASPs) is scarce.

**Methods:**

We reviewed 861 episodes of FN in outpatients with solid tumours, including 692 (80%) episodes with apparent clinical stability. We aimed to investigate the prognosis of this latter group and explore the possibility of stratifying it according to the presenting features. A case–control study was performed and the MASCC index was evaluated.

**Results:**

The rates of complications and bacteraemia in ASPs were 7.3% and 6.2%, respectively. The MASCC index yielded a low sensitivity to detect complications (36%). Prognostic factors were identified: ECOG performance status ⩾2, chronic bronchitis, chronic heart failure, stomatitis NCI grade ⩾2, monocytes <200 mm^−3^ and stress hyperglycaemia.

**Conclusion:**

A very simple assessment is useful to classify the patients with FN according to the risk of complications. A few additional variables may predict the clinical course of the patients. We additionally show that the MASCC index applied to this specific group has a low sensitivity to predict complications.

Febrile neutropenia (FN) is a common complication in cancer patients treated with chemotherapy. Although most of the cases are successfully solved with antibiotic treatment, a percentage of them can develop serious complications, and mortality is still significant in some settings (Sipsas *et al*, 2005). The prognostic models of Talcott and MASCC (see Table 1) were developed to predict the clinical outcome of patients with FN (Talcott *et al*, 1988; Klastersky *et al*, 2000). The latter model was validated several times and it is more accurate than Talcott's classification (Talcott *et al*, 1992; Uys *et al*, 2004). However, some problems still remain in the clinical setting. The derivation and validation of both models involved heterogeneous samples, with solid tumours, acute leukaemia and bone marrow transplantation (BMT), which differ in their baseline characteristics. Therefore, FN is managed differently in each particular scenario (Rossini *et al*, 1994; Nakagawa *et al*, 2009).

In the particular setting of Medical Oncology, the most common situations are moderate intensity chemotherapy, ambulatory setting, no profound immunosuppression and clinical stability at the onset (Talcott *et al*, 1988). In this situation, the rate of complications is around 9–15% according to the MASCC score (Klastersky *et al*, 2000, 2006). Although this group is less vulnerable, the introduction of home therapy and oral antibiotics can make the consequences of misclassification more relevant.

In addition, the patients enrolled in these studies showed a wide clinical spectrum, from fever without focus to multiorgan dysfunction (Talcott *et al*, 1988; Klastersky *et al*, 2000). As a result, the MASCC index includes some factors that define severe sepsis (Nguyen *et al*, 2006), even though they automatically point to high risk scenarios, thereby making the prediction irrelevant for changing medical decisions. Otherwise, the available literature regarding clinically stable patients is scarce (Moon and Chun, 2009). In this situation, there is also a rationale for incorporating biological variables into risk prediction (Jimeno *et al*, 2004; Uys *et al*, 2004). Therefore, it is still important to describe the clinical behaviour of more homogeneous samples of patients and, in particular, those who appear clinically stable at presentation. The primary aim of this study is to provide more data about the clinical features and prognosis of this specific population. The secondary objective is to explore the possibility of stratifying the group according to the presenting clinical features.

## Patients and methods

### Patients

The medical records of all adults (⩾18 years), outpatients, admitted between 1996 and 2004, with fever ⩾38 °C over 1 h, neutropenia ⩽500 mm^−3^ (or a count of ⩽1000 cells mm^−3^ with a predicted decrease to ⩽500 cells mm^−3^), chemotherapy and a solid tumour were reviewed. Patients treated with high-dose chemotherapy (HDC), including induction and intensification for acute leukaemia, BMT, the Magrath regimen and other induction treatments for Burkitt lymphoma, were excluded. The episode was considered valid if the management complied with current IDSA guidelines for FN (Hughes *et al*, 2002). Oral or ambulatory therapy was not offered. We additionally excluded the episodes in which ⩾10% of the variables tested were missing. In case of multiple episodes for one patient, only the first one was evaluated. The study protocol was approved by the hospital investigational review board, and informed consent was obtained from patients.

### Study design

*Initial risk stratification* We used objective criteria, such as the blood pressure, X-rays and blood tests, to classify the episodes into two categories: apparently stable patients (ASPs) and clearly unstable patients (CUPs). The CUP subgroup included those patients with any of these features at presentation: acute organ dysfunction, septic shock, hypotension, extensive infections (pneumonia, cellulitis >5 cm, typhlitis, meningitis and pyelonephritis) or any other acute comorbidity indicating hospital admission by itself. Patients without the attributes that characterised CUPs were considered ASPs. The medical records of the whole ASP group were reviewed to analyse the prevalence of complications that were not present at the diagnosis of FN, which comprised the triage and a basic workup (around 3 h).

*Potential risk factors in the ASP group* We designed a retrospective case–control study. Both cases and controls were obtained from the same base (ASP group). A case was defined as a patient with an unexpected serious complication after admission. These events were defined according to the MASCC criterion (Klastersky *et al*, 2000). All cases were included in the analysis. In patients with multiple complications, we considered the most severe one to define the case. A control was defined as a randomly selected patient who was not a case. The whole ASP subgroup was analysed to calculate the prevalence of complications and to provide a completely randomised selection of controls. For the multivariate study, we planned a ratio of 1 : 2 for cases and controls, plus additional 25% controls to compensate missing values. No matching techniques were used. Selection of prognostic variables was performed after reviewing the literature and considering their availability in the Emergency Room setting. We also omitted the variables that were not accessible at the onset. Burden of disease was evaluated as in Klastersky *et al* (2000). Constitutional syndrome was defined as the presence of asthenia, anorexia and loss of ⩾10% of body weight in the previous 3 months. As outlined in Table 2, most of the variables were dichotomic. For continuous variables, cutoffs were defined according to previous reports (Groeger *et al*, 1998) or, when unavailable, to clinically relevant or NCI toxicity scale-based cutoffs.

### Statistical considerations

For the univariate analysis, each potential risk factor was assessed using *χ*^2^-test or, when appropriate, Fisher exact test. Those variables with a *P*-value <0.20 were eligible for the multivariate analysis. The variables with ⩾10% of missing values (chills, heart rate, respiratory rate and fibrinogen) were excluded. Clinical redundancy and potential overlapping was also a reason for exclusion. The covariates finally included in the stepwise logistic regression model were: age, sex, ECOG, constitutional syndrome, COPD, albumin, monocytes, platelets, uraemia, stress hyperglycaemia, productive cough, abnormal X-rays without pneumonia, stomatitis, chronic heart failure, burden of disease, lung cancer and breast cancer. Statistical analyses were carried out with the SPSS 15.0 software (SPSS Inc., Chicago, IL, USA). All test were two-sided, and *P*-values <0.05 were considered significant.

## Results

**Figure 1:**
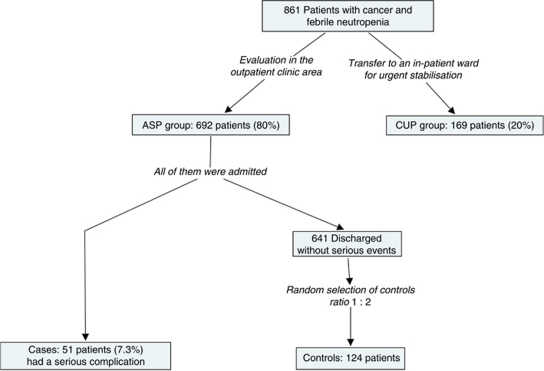
Flowchart summarising the study design.

The medical records of 861 valid episodes of FN were reviewed. We found that in 692 episodes (80%), there were neither clinical findings of instability nor a serious infection at the onset. Hence, according to the medical record, we classified them as ASPs (Figure 1). The other 169 episodes (20%) were classified as CUPs. The most frequent causes for classification as CUP were severe infections, but there were also complications unrelated to neutropenia, such as embolism or arrhythmia. We analysed what happened with ASPs during the hospital admission. Only 51 patients (7.3%) in the ASP group developed a serious complication, a rate in the range of low-risk MASCC group. The most frequent complications were acute respiratory failure (27 events), hypotension (25 events) and acute renal failure (11 events). Nine patients (1.3%) died after admission and the cause of death was clearly related to infection in seven of them (1%). Other complications were observed at a lower frequency (Table 3).

We observed a 6.2% rate of bacteraemia (43 episodes), which was lower than previously reported (Klastersky *et al*, 2007), a fact that we attributed to the selection of stable patients. We also found 22 cases (3.2%) of delayed lung infiltrates. These categories showed some overlap: 14 patients with bacteraemia and 7 patients with delayed lung infiltrates had serious complications. The main features of the patients and the febrile episodes are shown in Table 4. To determine which prognostic factors were relevant in ASPs, a case–control study was designed.

We performed a univariate analysis as shown on Table 5. The significant factors associated with complications were grouped in three categories: (1) baseline characteristics and comorbidity: age ⩾65 years, male, ECOG ⩾2, lung tumours, absence of breast cancer, COPD and chronic heart failure; (2) biological variables: albumin <3 mg dl^−1^, proteins <6.4 mg dl^−1^, uraemia >30 mg dl^−1^, stress hyperglycaemia, monocytes <200 mm^−3^ and platelets <1 00 000 mm^−3^; and (3) clinical characteristics of the febrile episode: stomatitis NCI grade ⩾2, productive cough, MASCC score <21 and constitutional syndrome. Interestingly, lung cancer showed an increased risk of complications (OR 3.43), mainly acute respiratory failure, whereas breast cancer was a protective factor (OR 0.34). In the multivariate analysis, six variables constituted independent risk factors: ECOG⩾2, COPD, chronic heart failure, stomatitis grade ⩾2, monocytopenia and stress hyperglycaemia (Table 6).

The absence of a validation set precluded a head-to-head comparison between our model and MASCC, but we analysed the distribution of the MASCC scores in the cases and controls (Table 5). We observed that 85% of the sample had a MASCC index ⩾21. We calculated the following parameters to detect complications: sensitivity 36%, specificity 94%, positive predictive value 32%, negative predictive value 94.9%, positive likelihood ratio 6, negative likelihood ratio 0.6 and correct classification 89%. The application of MASCC reduced the unexpected complications from 7.3 to 5.1%. These calculations suggest a poor performance of the model in stable patients, as 64% of the cases with complications have a MASCC score ⩾21. When the variables included in the MASCC score were analysed, we found that no patient had hypotension or previous invasive fungal infection, the in-patient status was 0% and the rate of dehydration was 7% (*n*=13), as expected. The remaining three variables occurred relatively more often: COPD was present in 20% (*n*=35), moderate burden of disease in 39% and age >60 yeas in 42% (*n*=71).

## Discussion

Febrile neutropenia is associated with significant morbidity and mortality in cancer patients. The MASCC index has been validated in the whole spectrum of FN and it is a precise tool for risk stratification (Klastersky *et al*, 2000). However, even in the low-risk group, around 10% of patients develop serious complications (Klastersky *et al*, 2006).

Our study was designed from a pragmatic point of view. We hypothesised that our target population was considerably homogeneous and differed from the samples in which Talcott and MASCC models were developed. We excluded the cases with acute leukaemia, HDC and BMT, which selected for patients with an anticipated short duration of neutropenia and a lower risk of complications. We also excluded in-patients because this prior status is considered a risk factor for complications according to MASCC and Talcott's models (Talcott *et al*, 1992). Moreover, an ultimate goal of prognostic models of low-risk FN is to identify a group of patients that can be suitable for home treatment, thus making the inclusion of in-patients problematic.

We also reasoned that a prognostic model is more helpful to make meaningful decisions in non-obvious situations, such as the assessment of ASPs with subtle clinical signs. According to this clinically oriented approach, we classified our patients into two subgroups that we called ASPs and CUPs, and focused in the prognosis and traits of the former ones. The ASP subgroup represents 80% of our practice in a Medical Oncology unit. We report that this simple classification yields a rate of complications in the range of the MASCC low-risk group. Furthermore, we found that 85% of ASPs were MASCC ⩾21 (low risk), but this model had a lower sensitivity in our population than previously reported. This low performance was suggested by a 64% of patients with complications showing a MASCC index ⩾21. We interpreted this finding as a result of the selection, which defined a different sample to that in which MASCC was validated. The MASCC scale includes eight parameters, and the definition of low risk is a score ⩾21. But when we applied the model to our sample, the reason for the low sensitivity became clear. There are no in-patients, hypotension and invasive fungal infections and the rate of dehydration is low. As a result, these factors become useless to differentiate some cases from others. Only three factors are left to make a prognostic distinction. Although moderate burden of disease, COPD and age >60 years occur more often, they are still relatively infrequent, and thereby they are of limited discriminatory help.

For our second objective, a retrospective case–control study was considered, as complications are rare events in ASPs. For example, the MASCC Study included 1351 patients, but the derivation set only contained 32 low-risk patients with unexpected complications (Klastersky *et al*, 2000). Our aim is not to make a comparison with MASCC, but to assess the possibility to further stratify the ASP group, according to the presenting clinical features. Our hypothesis is that to reduce uncertainty we need to select not only more homogeneous samples but also to consider different types of variables. In fact, the study proposes that a combination of comorbidities, biological variables and toxicities might improve the prognostic evaluation.

The independent variables were ECOG ⩾2, COPD, chronic heart failure, stomatitis grade ⩾2, monocytes <200 mm^−3^ and hyperglycaemia. These variables should not be adopted until further investigations could confirm the data and expose new sources of risk. However, all of them have a recognised biological substrate. Several studies have reported that a poor performance status is an adverse prognostic trait (Escalante *et al*, 1996; Sanz *et al*, 2002; Talcott *et al*, 2004; Klastersky *et al*, 2006). COPD is also a well-known risk factor in FN, as it is associated with serious bacterial infections and acute respiratory failure (Klastersky *et al*, 2000). The presence of stomatitis can predispose to bacteraemia (Bochud *et al*, 1994). Moreover, the inability to swallow is one of the causes that justify the hospitalisation (Rubenstein *et al*, 1993; Mullen *et al*, 1999; Talcott *et al*, 2004). Monocytopenia is linked with bacteraemia in children with FN (Rackoff *et al*, 1996), and early monocytopenia after chemotherapy is correlated with neutropenia (Oguz *et al*, 2006). Finally, hyperglycaemia is common in critically ill patients, in which it is a well-known risk factor of complications (Dandona *et al*, 2003). Moreover, a randomised trial has shown that intensive insulin therapy reduces morbidity and mortality in this setting (Van den Berghe *et al*, 2001). We believe that the evaluation of biological variables is interesting in FN. Particularly, some reports have found that acute-phase reactants are useful as adjuvants to the clinical models (Kern *et al*, 2001; Nijhuis *et al*, 2005). Finally, our model has some other limitations beyond the requirement of validation. The selection of variables was based on easy availability, which excluded some potentially informative factors. Furthermore, all our patients were admitted to the hospital and received intravenous treatment, which might compromise the applicability of the model in the ambulatory setting.

As a conclusion, we have shown that a very simple assessment (ASP *vs* CUP) can classify cancer patients with FN according to the risk of complications. A few additional variables predict the clinical course of this population. Finally, the MASCC index applied to this specific group has a low sensitivity to predict complications.

## Figures and Tables

**Table 1:** The MASCC index score

**Category**	**Weight**
Burden of illness: no or mild symptoms	5
No hypotension	5
No chronic obstructive pulmonary disease	4
Solid tumour or no previous invasive fungal infection	4
Outpatient status	3
Burden of disease: moderate symptoms	3
No dehydration	3
Aged <60 years	2

Abbreviation: MASCC=Multinational Association of Supportive Care in Cancer.

The maximum theoretical score is 26. A MASCC score ⩾21 identifies low-risk patients with a positive predictive value of 91%, specificity of 68% and sensitivity of 71% (Klastersky *et al*, 2000).

**Table 2:** Variables tested as potential risk factors in febrile neutropenia outcome

**Variables**	**Categories**
*Baseline characteristics and medical history*	
Age, years	⩾65, <65
Sex	Male, female
Surgery in the previous month	Yes, no
ECOG performance status	⩾2, <2
Antibiotics in the previous month	Yes, no
Constitutional syndrome	Yes, no
COPD	Yes, no
Chronic heart failure	Yes, no
Diabetes mellitus	Yes, no
Lung cancer	Yes, no
Lymphoma	Yes, no
Breast cancer	Yes, no
TNM stage	IV, I–III
Prophylactic GCSF	Yes, no
Adjuvant chemotherapy	Yes, no
	
*Laboratory results*	
Albumin, g dl^−1^	<3, ⩾3
Total proteins, g dl^−1^	<6.4, ⩾6.4
LDH, mU ml^−1^	⩾475 , <475
Monocytes at the onset, mm^−3^	<200, ⩾200
Platelets at the onset, mm^−3^	<1 00 000, ⩾1 00 000
Haemoglobin at the onset, g dl^−1^	⩽9, >9
Neutrophils at the onset, mm^−3^	⩽100, >100
Fibrinogen, mg dl^−1^	⩾450 , <450
Uraemia, mg dl^−1^	⩾30 mg, <30
Stress hyperglycaemia^a^	Yes, no
	
*Characteristics of the febrile neutropenia episode*	
Productive cough	Yes, no
Urinary infection	Yes, no
Fever of unknown origin	Yes, no
Abnormal X-ray without pneumonia at the onset	Yes, no
Heart rate ⩾91	Yes, no
Respiratory rate ⩾21	Yes, no
MASCC score	<21, ⩾21
Burden of disease at the onset	Moderate, low
Dehydration	Yes, no
Chills	Yes, no
Temperature at the onset, °C	⩾39, <39
Stomatitis NCI grade ⩾2	Yes, no

Abbreviations: COPD=chronic obstructive pulmonary disease; ECOG=Eastern Cooperative Oncology Group; GCSF=granulocyte-colony stimulating factor; LDH=lactate dehydrogenase; MASCC=Multinational Association of Supportive Care in Cancer; NCI=National Cancer Institute; TNM=tumour-node-metastasis.

a Stress hyperglycaemia: ⩾121 mg dl^−1^ (⩾250 mg m^−2^ in diabetic patients).

**Table 3:** Distribution of acute serious complications

**Complication**	***n* (%)** a
Acute respiratory failure	27 (28)
Hypotension	25 (26)
Acute renal failure	11 (11)
Death	9 (9.5)
Altered mental status	6 (6.3)
Acute heart failure	4 (4.2)
Serious bleeding	4 (4.2)
Acute abdomen	3 (3.1)
Arrhythmia	2 (2.1)
Disseminated intravascular coagulation	2 (2.1)
Unstable angina	1 (1)
Total	94

a Number of complications. Multiples complications for one patient are shown here.

**Table 4:** Main features of the patients and the febrile episodes

**Characteristic**	***N* (%)**
ECOG performance status 0–1	138 (78)
	
*Tumours*	
Breast	53 (30)
Lung	41 (23.4)
Lymphoma	31 (18)
Sarcoma	13 (7.5)
Colorectal	7 (4)
Other solid tumours	30 (17)
	
*Treatment setting*	
Adjuvant	33 (19)
Neoadjuvant	15 (8)
Advanced disease/palliative	96 (55)
Other	31 (18)
	
TNM stage IV	96 (55)
MASCC⩾21	144 (85)
	
*Cause of the fever*	
Fever of unknown origin	80 (37)
Clinically confirmed infection	84 (39)
Microbiologically confirmed infection	51 (24)
	
Days with neutropenia ⩽500 mm^−3^	3 (range 1–8)
Length of admission	7.4 days
Rate of unexpected complications	7.3
	
*Rate of bacteraemia*	6.2
Percentage of Gram negative in blood cultures	65
	
Median age, years	55 (range 13–85)

Abbreviation: ECOG=Eastern Cooperative Oncology Group.

**Table 5:** Predictive factors for serious complications in the univariate analysis

**Variables**	**Cases, *n* (%)**	**Controls, *n* (%)**	**OR**	**95% CI**	***P*-value**
*Baseline characteristics and medical history*					
Age, years					
<65	30 (58.8)	93 (78.2)	2.5	1.23–5.07	0.010
⩾65	21 (41.2)	26 (21.8)			
					
Sex					
Female	17 (33.3)	74 (62.2)	3.28	1.65–6.55	0.001
Male	34 (66.7)	45 (37.8)			
					
Surgery in the previous month					
No	47 (92)	108 (88.5)	0.6	0.2–2.1	0.6
Yes	4 (8)	14 (11.5)			
					
ECOG performance status					
<2	32 (62.7)	106 (89.1)	4.84	2.1–10.86	<0.0001
⩾2	19 (37.3)	13 (10.9)			
					
Antibiotics in the previous month					
No	32 (62.7)	90 (73.7)	1.6	0.83–3.35	0.14
Yes	19 (37.3)	32 (26.3)			
					
Constitutional syndrome					
No	26 (51)	93 (78.2)	4.43	1.7–6.92	<0.0001
Yes	25 (49)	26 (21.8)			
					
Chronic bronchitis					
No	28 (54.9)	107 (89.9)	7.32	3.25–16.5	<0.0001
Yes	23 (45.1)	12 (10.1)			
					
Chronic heart failure					
No	42 (82.4)	115 (97.5)	8.21	2.12–31.8	<0.0001
Yes	9 (17.6)	3 (2.5)			
					
Diabetes mellitus					
No	48 (94.1)	117 (95)	1.46	0.3–6.3	0.69
Yes	3 (5.9)	5 (5)			
					
Lung cancer					
No	30 (58.8)	99 (83.2)	3.43	1.66–7.23	0.01
Yes	21 (41.1)	20 (16.8)			
					
Breast cancer					
No	43 (84.3)	74 (62.2)	0.34	0.13–0.7	0.04
Yes	8 (15.7)	45 (37.8)			
					
Lymphoma					
No	42 (82.3)	100 (81.9)	0.9	0.41–2.29	0.57
Yes	9 (17.7)	22 (18.1)			
					
TNM stage					
I–III	20 (39.2)	59 (48.3)	1.45	0.7–2.8	0.31
IV	31 (60.8)	63 (51.7)			
					
Prophylactic GCSF					
No	44 (86.2)	101 (82.7)	0.84	0.3–2.15	0.72
Yes	7 (13.8)	19 (17.3)			
					
Adjuvant chemotherapy					
No	43 (84.3)	95 (77.8)	0.65	0.2–1.55	0.4
Yes	8 (15.7)	27 (22.2)			
					
*Laboratory results*					
Albumin, mg dl^−1^					
⩾3	29 (59.2)	91 (78.4)	2. 51	1.22–5.16	0.011
<3	20 (40.8)	25 (21.6)			
					
Total proteins, mg dl^−1^					
⩾6.4	7 (14.6)	59 (51.3)	6.17	2.55–14.8	<0.0001
<6.4	41 (85.4)	56 (58.7)			
					
LDH, mU ml^−1^					
<475	30 (58.9)	81 (66.3)	1.42	0.7–2.9	0.35
⩾475	18 (41.1)	34 (33.6)			
					
Haemoglobin at the onset, gr dl^−1^					
⩾9	39 (76.4)	102 (83.6)	1.6	0.7–3.7	0.27
<9	12 (23.6)	19 (16.4)			
					
Neutrophils at the onset, mm^−3^					
⩾100	28 (54.9)	71 (58.1)	1.1	0.6–2.2	0.73
<100	23 (45.1)	50 (41.9)			
					
Monocytes at the onset, mm^−3^					
⩾200	14 (27.5)	62 (52.1)	2.87	1.41–5.86	0.003
<200	37 (72.5)	57 (47.9)			
					
Platelets at the onset, mm^−3^					
⩾1 00 000	20 (39.2)	80 (67.8)	3.26	1.65–6.45	0.001
<1 00 000	31 (60.8)	38 (32.2)			
					
Uraemia, mg dl^−1^					
⩽30	19 (43.2)	94 (80.3)	5.37	2.53–11.3	<0.0001
>30	25 (56.8)	23 (19.7)			
					
Stress hyperglycaemia^a^					
No	23 (47.9)	94 (80.3)	4.42	2.14–9.19	<0.0001
Yes	25 (52.1)	23 (19.7)			
					
*Characteristics of the febrile neutropenia episode*					
Abnormal X-ray without pneumonia					
No	19 (37.3)	88 (73.9)	4.78	2.37–9.62	<0.0001
Yes	32 (62.7)	31 (26.1)			
					
Stomatitis NCI grade					
<2	31 (60.8)	94 (79.7)	2.52	1.23–5.18	0.010
⩾2	20 (39.2)	24 (20.3)			
					
MASCC					
⩾21	32 (64)	112 (94)	7.9	3–20.5	<0.0001
<21	18 (36)	7 (6)			
					
Urinary infection					
No	46 (90.1)	112 (91.8)	1.2	0.39–3.7	0.7
Yes	5 (9.9)	10 (8.1)			
					
Productive cough					
No	34 (66.7)	111 (94.1)	7.92	3.03–20.7	<0.0001
Yes	17 (33.3)	7 (5.9)			
					
Burden of disease					
Low	18 (35)	107 (69)	4.3	2.15–8	<0.0001
Moderate	33 (65)	48 (31)			

Abbreviations: CI=confidence interval; ECOG=Eastern Cooperative Oncology Group; GCSF=granulocyte-colony stimulating factor; LDH=lactate dehydrogenase; NCI=National Cancer Institute; OR=odds ratio; TNM=tumour-node-metastasis.

a Stress hyperglycaemia: ⩾121 mg dl^−1^ (⩾250 mg m^−2^ in diabetic patients).

**Table 6:** Predictive factors for serious complications in the multivariate analysis

**Variable**	**OR**	**95% CI**	***P*-value**
ECOG, ⩾2	2.40	1.03–5.55	0.041
Chronic bronchitis	4.45	1.95–10.17	<0.0001
Chronic heart failure	6.47	1.60–26.15	0.009
Stomatitis grade, ⩾2	2.59	1.15–5.81	0.02
Monocytes, <200 mm^−3^	2.29	1.04–5.07	0.04
Stress hyperglycaemia	3.06	1.43–6.54	0.004

Abbreviations: CI=confidence interval; ECOG=Eastern Cooperative Oncology Group; OR=odds ratio.
